# Editorial: Use of small peptides in the treatment of inflammatory diseases

**DOI:** 10.3389/fphar.2022.1090014

**Published:** 2022-11-24

**Authors:** Sylvain Chemtob, Yvonne Rosenstein, Constance Auvynet

**Affiliations:** ^1^ Departments of Pediatrics and Ohptalmology/Pharmacology, University of Montreal, Montreal, QC, Canada; ^2^ Instituto de Biotecnología, Universidad Nacional Autónoma de México, Cuernavaca, Mexico

**Keywords:** peptide, Inflammatory diseases, Immunomodulation, Diagnostic tools, dual activity

Inflammation is essential in the resolution of infection or tissue damage. However, excessive inflammation, which sometimes lasts for years, leads to numerous inflammatory disorders such as cardiovascular, neurodegenerative, or gastrointestinal diseases, cancer or diabetes mellitus, or even infections. Small peptides are promising therapeutic tools as they have been shown to present higher specificity (especially peptides with allosteric properties) and binding affinity than small molecules, and reduced immunogenicity and toxicity than biologics. Moreover, some of them have a dual—anti-inflammatory and antimicrobial—activity.

The source of therapeutic peptides is almost inexhaustible as they can have natural or designed origins. Interestingly, peptidic compounds entering clinical trials are more susceptible to approval. In the last 20 years, novel designs, delivery strategies, and improvements in peptide production and modification have led to a total of 33 approved peptide drugs, and more than 170 peptides are in clinical trials (Wang et al., 2022). In their comprehensive review focused on the state of clinical trials on non-cancer dermatological biologics in China, Zhu et al. show that the number of dermatological biologic trials in China surged between 2016 and 2020, primarily driven by psoriasis trials.

To control undesirable inflammation with reduced side effects, it is desirable to target a particular signaling pathway, cell type, tissue, or organ, without increasing susceptibility to infections or diseases secondary to the treatment. Additional to those considerations, the development of promising immunomodulatory peptide candidates needs to contemplate when and how they will best contribute to inflammation resolution. As a first step to comply with those requirements, it is necessary to elucidate the origin and potential functions of the diverse immune cells present in a tissue at a given time. Especially, macrophages’ multiple functions in wounds or infections, such as the induction and resolution of inflammation, the removal of apoptotic cells, cell proliferation, and tissue repair, but also promote excessive inflammation, leading to various disorders, make of macrophages a promising therapeutic target to control the balance between necessary and excessive inflammation. In their article, Golden et al. investigated the origin of the different macrophage populations present in the acute lung injury and the resolution phases in the intratracheal bleomycin mouse model. In this model of acute inflammation, tissue-resident macrophages in the lung are downregulated in the acute phase of inflammation before being regenerated in the resolving phase of inflammation. In contrast, monocyte-derived macrophages are recruited to the lung during the acute phase of inflammation and are responsible for the release of iNOS and excess inflammation that leads to acute lung injury. These results stress the concept that depending on the inflammatory stage, macrophages exhibit a particular phenotype and activation state, making individual populations of macrophages a promising target to modulate different inflammation stages.

In another study, Lee et al. developed a mouse leukocyte migration assay using a lower uterine extract chemoattractant that could be used as a diagnostic tool for pre-term birth. With this test, as a proof of concept, the authors showed that IL-1beta stimulated pre-term birth by activating neutrophils, leading to increased uterine and fetal brain activation. This IL-beta stimulation was inhibited by the rytvela peptide, an allosteric antagonist of the IL-1 receptor that selectively inhibits the IL-1R signaling pathway down the mitogen-activated protein kinase/p38, but not the nuclear factor kappa B one, enabling immunosurveillance. Again, this work underscores how a better appreciation of the biological role of potential therapeutic targets is fundamental in the search and use of new immunomodulatory candidates.

Another challenge in controlling undesirable inflammation is to direct the therapeutic peptide to a specific tissue or organ at the right moment. One of the drawbacks in the use of peptides as therapeutic agents is their poor membrane permeability, pinpointing the drug delivery system as key to the success of therapy. Different strategies have been developed in the past years, such as co-formulation with permeation enhancers or implantable pumps (Farra et al., 2012; Knudsen et al., 2019). Due to their biochemical properties and skin-mimicking structure, hydrogels loaded with bioactive peptides are a promising strategy for wound healing and tissue restoration. Hao et al. show that using a chitosan/alginate hydrogel combined with short-chain peptides isolated from the velvet antler blood (a remedy used in traditional Chinese medicine) contribute to rapid wound healing and skin repair. Indeed, the biochemical and biophysical properties of the hydrogel loaded with the velvet antler blood peptides exhibit a combined biological activity, i.e., antibacterial capacity and excessive inflammation inhibition, resulting in local skin repair.

Another limitation in peptide drug development is their poor *in vivo* stability. Due to their composition and structure, small peptides are susceptible to being degraded by various enzymes and rapidly eliminated *in vivo*. In recent years significant breakthroughs in chemical peptide synthesis, but also rational design, use of all-D peptides and phage display have been developed to solve this limitation. In their article, to overcome the limitation of poor *in vivo* stability, Luo et al. used a strategy based on the inhibition of endogenous enzymes. Using a model of mouse colitis where the anti-inflammatory role of enkephalins contributes to lessen the inflammation of the colon, the authors showed that central administration of human opiorphin, the natural inhibitor of enkephalinase, suppresses the activity of natural endo- and amino-peptidases, thus favoring higher levels of enkephalin in the serum and the improvement of the colitis.

In conclusion, this research topic highlights some of the key points to be considered for the development of succesfull peptide drugs: the identification of the therapeutic targets and understanding their mechanisms of action, and the improvement of *in vivo* stability and delivery.

## References

[B1] Farra R. Sheppard N. F. McCabe L. Neer R. M. Anderson J. M. Santini J. T. (2012). First-in-human testing of a wirelessly controlled drug delivery microchip. Sci. Transl. Med. 4, 122ra21. 10.1126/scitranslmed.3003276 22344516

[B2] Knudsen L. B. Lau J. (2019). The discovery and development of liraglutide and semaglutide. Front. Endocrinol. 10, 155. 10.3389/fendo.2019.00155 PMC6474072 31031702

[B3] Wang L. Wang N. Zhang W. Cheng X. Yan Z. Shao G. (2022). Therapeutic peptides: Current applications and future directions. Signal Transduct. Target. Ther. 147 (1), 48. 10.1038/s41392-022-00904-4 PMC8844085 35165272

